# Supplementary material for: Different Associations between Tonsil Microbiome, Chronic Tonsillitis, and Intermittent Hypoxemia among Obstructive Sleep Apnea Children of Different Weight Status: A Pilot Case-Control Study
Source: J Pers Med. 2021 May 28;11(6):486. doi: 10.3390/jpm11060486 (PMC8227284; doi:10.3390/jpm11060486)
Supplement: Supplementary file 1 [file jpm-11-00486-s001.zip › jpm-1229152-supplementary.pdf]

Journal of
Personalized
Medicine

MDPI

Supplementary Materials

Table S1. Patient characteristics, polysomnography variables, and blood pressures of the over-weight and under-weight subgroups.

Variables	Over-weight subgroup	Under-weight subgroup	*p*-Value[1]
	n = 30	*n* = 3	
**Patient characteristics**			
Age (years)	7.0 (6.0–10.0)	8.0 (5.0–)	> 0.999
Male sex, *n* (%)	25 (83%)	3 (100%)	> 0.999
Chronic tonsillitis, *n* (%)	6 (20%)	0 (0%)	> 0.999
Allergic rhinitis, *n* (%)	19 (63%)	3 (100%)	0.534
BMI (kg/m^2) z-score	2.10 (1.40–2.44)	-7.60 (-7.45–)	< 0.001 *
Tonsil size	3 (3–4)	4 (3–)	0.260
ANR	0.73 (0.63–0.83)	0.74 (0.67–)	0.571
OSA-18 score	80 (69–94)	77 (73–)	0.614
**Polysomnography variables**			
AHI (events/h)	9.6 (5.1–26.3)	6.1 (6.0–)	0.571
RDI (events/h)	12.1 (7.3–29.7	13.1 (6.0–)	0.791
ODI (events/h)	8.5 (3.4–23.2)	3.2 (1.8–)	0.100
Mean SpO_2 (%)	97 (96–98)	92 (91–)	0.082
Minimal SpO_2 (%)	89 (83–91)	91 (88–93)	0.070
N1 stage	14 (6–21)	10 (9–)	0.837
N2 stage	37 (31–45)	41 (37–)	0.491
N3 stage	28 (23–31)	19 (17–)	0.260
REM stage	18 (12–22)	21 (21–)	0.149
**Blood pressure variables**			
Systolic BP, mmHg	111 (100–121)	85 (61–)	0.188
Diastolic BP, mmHg	66 (61–74)	64 (46–)	0.571
Systolic BP percentile (%)	85 (66–91)	17 (8–)	0.260
Diastolic BP percentile (%)	75 (55–87)	71 (27–)	0.701

Note: Data are summarized as median (interquartile range) or *n* (%) as appropriate. Abbreviations: AHI, apnea–hypopnea index; ANR, adenoidal-nasopharyngeal ratio; BMI, body mass index; BP, blood pressure; ODI, oxygen desaturation index; OSA, obstructive sleep apnea; RDI, respiratory disturbance index; REM, rapid eye movement; SpO2, pulse oxygen saturation. [1] Data were compared using the Mann-Whitney *U* test for continuous variables, and the chi-square test for categorical variables. * Significant differences $p < 0.05$.
